# Synthesis, Properties of Biodegradable Poly(Butylene Succinate-*co*-Butylene 2-Methylsuccinate) and Application for Sustainable Release

**DOI:** 10.3390/ma12091507

**Published:** 2019-05-09

**Authors:** Jiarui Han, Jiaxin Shi, Zhining Xie, Jun Xu, Baohua Guo

**Affiliations:** Key Laboratory of Advanced Materials of Ministry of Education of China, Department of Chemical Engineering, Tsinghua University, Beijing 100084, China; chaxiangmeimei@163.com (J.H.); shijx13@tsinghua.org.cn (J.S.); xiezhining@126.com (Z.X.)

**Keywords:** biodegradable copolyesters, crystallinity, degradation property, pesticide-loaded microparticles, pesticide release

## Abstract

A novel biobased and biodegradable polyester, i.e., poly(butylene succinate-*co*-butylene 2-methylsuccinate) (P(BS-BMS)) was synthesized by succinic acid (SA), 2-methylsuccinic acid (MSA), and 1,4-butanediol (BDO) via a typically two-step esterification and polycondensation procedure. The chemical structure and macromolecular weight of obtained copolymers were characterized by ^1^H NMR, ^13^C NMR, and GPC. The melting temperature and degree of crystallinity were also studied by DSC, and it was found that the values were gradually decreased with increasing of MSA content, while the thermal stability remained almost unchanged which was tested by TGA. In addition, the biodegradation rate of the P(BS-BMS) copolymers could be controlled by adjusting the ratio of SA and MSA, and such biodegradability could make P(BS-BMS) copolymers avoid microplastic pollution which may be brought to the environment for applications in agricultural field. When we applied P(BS-BMS) copolymers as pesticide carriers which were prepared by premix membrane emulsification (PME) method for controlling Avermectin delivery, an improvement of dispersion and utilization of active ingredient was obviously witnessed. It showed a burst release process first followed by a sustained release of Avermectin for a long period, which had a great potential to be an effective and environmental friendly pesticide-release vehicle.

## 1. Introduction 

Nowadays, in view of the threat of environmental pollution and resource shortage to sustainable ecological construction, fully biobased and biodegradable materials becomes an urgent issue in both academic and industrial fields [1,2,3,4]. As a result of this increasing attention, numerous renewable polymers including thermoplastic [5], thermoset [6,7], and vitrimers [8,9] with multiple complex performance have been developed in order to replace traditional petroleum-based materials. Among these sustainable polymers, in view of the excellent biodegradability [10,11,12,13,14,15,16,17], poly(butylene succinate) (PBS) and its copolymers [18,19,20] are widely used in different fields such as agriculture [21,22], food packages [23], and hygiene products [24]. Bi, et al. [25] investigated the relationship between the rate of enzymatic degradation and the ratio of butanediol to hexanediol in the poly(butylene succinate-*co*-hexamethylene succinate). This kind of copolymers shows a great potential as a sustainable substitute with tunable enzymatic degradation in agriculture and packaging industries. Besides the biodegradable property, for meeting the different requirements of application, the chemical structures of PBS are usually modified by copolymerization with a third monomer such as terephthalic acid [26,27] (diacids), hexanediol [28] (diols), and other functional monomers [29,30]. Generally, the crystallization property of the polymer is a crucial factor, which can be flexibly controlled by copolymerization [31,32]. Zhang, et al. [33] discussed the interplay between the Diels–Alder reaction and crystallization in poly(2,5-furandimethylene succinate)-b-poly(butylene succinate) (PFS-PBS) multiblock copolyesters. The PFS-PBS copolymers can be adjusted flexibly from soft to rigid materials by controlling the PFS content. Zheng, et al. [34] demonstrated a multiblock copolymer composed of poly(butylene succinate) (PBS) and poly(butylene fumarate) (PBF) which could provide various reactive sites on the main chain for further modification. It showed an isomorphic cocrystallization phenomenon in PBS-PBF block copolymers. As an economically viable renewable build unit, itaconic acid (IA), and its derivatives get more exploration [35,36,37]. Trotta, et al. [3] reported a novel thermoplastic which was almost completely derived from IA while high atom economies and mild process conditions for reaction procedure indicates “green process”. 2-methylsuccinate acid (MSA) which can be simply produced by catalytic hydrogenation of IA owns similar structure with SA [38]. Wu, et al. [39] declared a novel aliphatic polyester synthesized from MSA and BDO, which displayed desirable mechanical property and superior migration resistant property as bio-based polymeric plasticizer. The similar structure leads to the potential to regulate the crystallinity of PBS chains by introduction of MSA. Since it can flexibly adjust the properties of synthetic copolyesters like crystallization, stability, and biodegradation by changing the type of copolymerization units and the copolymerization ratio to expand the material application fields, synthetic copolyesters have been taken close attention of many researchers.

The use of fully biobased and biodegradable polymers in agriculture field has increased dramatically in recent years throughout the world due to possessing equal performance while not putting pressure on the environment like white pollution [40,41,42,43,44]. For example, numerous degradable polymers including natural ones like starch and synthetic ones like poly(butylene adipate-*co*-terephtalate) (PBAT), polylactic acid (PLA), poly(3-hydroxybutyrate) (P3HB) and poly(butylene succinate) (PBS) have been applied rather than polyethylene as agriculture mulch [45,46]. Zhang, et al. [29] introduced a kind of UV absorber 2-hydro-4-(2,3-epoxypropoxy) benzophenone (HEPBP) into PBS chains via polycondensation. This functional PBS owned excellent UV protection effect as agriculture mulch use. Lubkowski, et al. [47] illustrated a controlled-release multicomponent fertilizer coated with a biodegradable copolymer consisting of poly(butylene succinate) and a butylene ester of dilinoleic acid. A conceptual model describing the release of nutrient through the coated layer was presented. In addition, the protection and efficient use of pesticides is another challenge for agriculture [48,49,50,51]. Traditional formulations usually use a large amount of organic solvent and dispersants in order to enhance the dispersibility of hydrophobic pesticide components, consequently posing a serious threat to food safety and ecological environment [52]. Furthermore, owing to the instability properties of pesticides and traditional pesticide formulations which include photolysis, evaporation, and surface slip, the active ingredient content in conventional formulations is rapidly consumed, falling below the effective level after an initial burst release. In order to improve the shortcomings of traditional pesticide formulations, researchers have conducted extensive research on pesticide carriers. Jia, et al. [53] demonstrated a novel polydopamine microcapsule containing Avermectin prepared by in situ emulsion interfacial polymerization. Experimental results showed the excellent sustained-release of active ingredient and good adhesive performance on plants leaves. Li et al. [54] illustrated the preparation of uniform starch microparticles containing Avermectin via premix membrane emulsification (PME) method. The effects of microparticle size, Av content, and carrier morphology on release performance were investigated in this work. PME is well known as a mild technique to produce stable emulsion with highly uniform droplets while expending relatively low energy input. Therefore, it is widely used in medicine, painting, cosmetics, and other industries [55,56,57,58,59]. 

Herein, we applied a novel kind of biobased and biodegradable copolymer poly(butylene butylene succinate-*co*-2-methylsuccinate) (P(BS-BMS)) in pesticide release field which has not been reported. We introduced MSA monomer instead of part of SA unit in the normal polycondensation process to regulate the chain structure in order to tune the properties, especially crystallization and biodegradability of copolymers, which have a significant impact on evaluation of pesticide carrier like drug loading, releasing rate, and environmental pollution. Furthermore, a model drug Avermectin (Av) was encapsulated in the uniform P(BS-BMS) microparticles by PME method. The effect of copolymer crystallinity on the morphology of Av-loaded microparticles and performance of Av release was revealed. Based on such the environmental-friendly property and optimal drug loaded performance, P(BS-BMS) is a kind of promising materials as pesticide delivery carrier in the pesticide application field.

## 2. Experimental Section

### 2.1. Materials

2-methylsuccinic acid (MSA, >99.0%) was provided by Shenzhen VTOLO Industrial Co., Ltd, Shenzhen, China. 1,4-butanediol (BDO, >99.5%) and succinic acid (SA, purity >99.5%) were supplied by Tianjin Jinke Fine Chemical Research Institute and Anqing Hexing Chemicals Co., Ltd. (Anqing, China), respectively. Poly(vinyl alcohol) (PVA1788, 87.0–89.0% hydrolyzed with an average *M*_w_ of 30,000–70,000) was purchased from Aladdin Industrial Corporation. Avermectin (Av, Qilu Pharmaceutical (Inner Mongilia) Co., LTD., Inner Mongolia, China, 95%) and Avermectin EC (Hebei Weiyuan Chemical Co., Ltd, Shijiazhuang, China, 5 wt%) were obtained. Methanol (HPLC Grade, 99.9%) was supplied by J&K Scientific Ltd. (Shanghai, China). Other reagents and solvents (AR grade) were used directly without any purification.

### 2.2. Synthesis of P(BS-BMS) Copolyesters

P(BS-BMS) copolyesters were synthesized through a two-stage process including direct esterification and melt polycondensation. First, predetermined amount of SA, MSA, and BDO were added into a three-necked flask and heated to 135 °C with continuing mechanical agitation in N_2_ atmosphere. Then, the temperature was raised to 225 °C gradually step by step in order to make the esterification reaction sufficiently. When there was no more water produced, the temperature was decreased to 150 °C and a certain amount of catalyst tetrabutyl titanate was added with stirring for 5 min followed by the polycondensation proceeded at a pressure <100 Pa for half an hour. Thereafter, maintaining the vacuum, the temperature was raised to 230 °C to continue the reaction for 2–4 h until a Weissenberg effect was observed. After the polymerization process, the samples were dissolved in chloroform, precipitated in cold methanol and then dried in vacuum oven at 40 °C for 24 h. The copolymers thus synthesized were denoted as P(BS-BMS)x%, where x% represents the molar percentage of MSA based on all diacid monomers, that is, x% = MSA/(SA + MSA) × 100%. P(BS-BMS)10%, 20%, 30%, 50%, and pure PBS were prepared via this method.

### 2.3. Preparation of the Av-Loaded P(BS-BMS) Microspheres

The typical process for preparing the Av-loaded microsphere and blank microspheres by premix membrane emulsification (PME, National Engineering Research Center for Biotechnology) is briefly described as follows. First of all, 4 mL of chloroform solution containing a certain amount of P(BS-BMS) copolyester and Av active compound was poured into a large volume of 0.5 wt% PVA aqueous solution under mechanical stirring to prepare a primary emulsion. Then the primary emulsion was forced through the Shirasu Porous Glass membrane (SPG membrane) three times at a N_2_ pressure of 0.25 MPa. The emulsion was obtained and evaporated in 30 °C water bath oscillator overnight. Finally, the solid microspheres were collected through centrifugation and washed 3 times with deionized water. The washed microspheres were freeze-dried for 24 h to obtain solid powder.

### 2.4. Polymer Characterizations

The ^1^H NMR and ^13^C NMR spectra were obtained by a fully automated NMR spectrometer (JEOL, ECA-600M, Tokyo, Japan), using deuterated chloroform as solvent. The molecular weight and dispersity (Đ) were measured by gel permeation chromatography (GPC) (Waters 1515, Waters, Milford, MA, USA) equipped with a differential-refractometer detector. The measurements were taken at 35 °C and the chloroform was used as the eluent with a flow rate of 1.0 mL/min. The number and weight average molecular weight was calculated by using a calibration curve with monodisperse polystyrene as standards. 

Differential scanning calorimetry (DSC, TA equipment, Q 2000, TA Instruments, New Castle, DE, USA) was used to determine *T*_m_ values and analyze the crystallization property of copolymer samples with different MSA percentage. The samples (3–5 mg) were sealed in aluminum crucibles and first heated to 150 °C followed by keeping isothermal for 10 min to eliminate the thermal and reaction history under N_2_ atmosphere. After that, the samples were cooled to −50 °C at a rate of −10 °C/min, and reheated to 150 °C at a rate of 10 °C/min. The thermal decomposition behavior was performed with a Shimadzu DTG-60 thermo gravimetric analyzer (Kyoto, Japan). Each sample (8–10 mg) was heated from 30 °C to 600 °C at 10 °C/min in N_2_ atmosphere with a flow rate of 40 mL/min. The crystal structure of P(BS-BMS) copolymers was measured by wide-angle X-ray diffraction (WAXD, BrukerD8 Advance, Bruker, Hamburg, Germany) in powder diffraction mode. The samples were melted and prepared to films for testing. Scanning was performed with 2θ from 5 to 40° with a step of 0.02°.

### 2.5. In Vitro Degradation Test

P(BS-BMS) copolymer films were prepared by hot pressing method and then stored at room temperature at least 24 h. The 2 mg/mL enzyme degradation test solution was prepared by dissolving a certain amount of amano lipase from Pseudomonas fluorescens in the phosphate buffer saline (pbs, pH 7.2–7.4), and the enzyme-free pbs solution was used as the controls. The film samples (10 mm × 10 mm × 0.2 mm) were immersed in 10 mL degradation solutions and incubated at 37 °C with continuous shaking at 100 rpm. The films were removed from the solution every 2 days, rinsed with deionized water and freeze-dried for 12 h. The degradation solution was refreshed after each measurement. The degradation degree was quantified as weight loss and the surface morphology of these samples were observed using field emission scanning electron microscopy (FESEM, JSM-7401, JEOL, Tokyo, Japan). For SEM test, an accelerating voltage of 3 kV was applied, and the sample was sputtered with platinum prior to the test.

### 2.6. Characterizations of Av-Loaded Microspheres

The morphology of Av-loaded microspheres was also observed by FESEM (JSM-7401). Sample preparation and operating conditions are similar to the above mentioned. The particle size and size distribution were obtained by counting statistics of SEM graphs, using Nano Measurer software (Version 1.2.0.0). And the particle size distribution was calculated according to the following equations:

(1) $$D_{n}=\sum n_{i}D_{i}/\sum n_{i}$$

(2) $$D_{w}=\sum n_{i}D_{i}{}^{4}/\sum n_{i}D_{i}{}^{3}$$

(3) $$PDI=D_{w}/D_{n}$$

The crystal structure of Av and Av-loaded microspheres was measured by wide-angle X-ray diffraction in powder diffraction mode. The samples were ground and placed in sample cell for testing. Scanning was performed with 2θ from 5 to 40° with a step of 0.02°. For the determination of the drug loading content, briefly, approximately 5 mg Av-loaded microspheres were fully ground and dispersed in 8 mL methanol, then sonicated for a sufficient period of time to ensure that all active ingredients were leached. Finally, the dispersion was filtered to form a clear Av solution before analysis. The loading content (LC) and entrapment efficiency (EE) of Av were measured by high-performance liquid chromatography (HPLC, Shimadzu 16, Kyoto, Japan) using a C18 column with column temperature of 30 °C. The methanol/water (90/10) mixture was served as the elution with a rate of 1 mL/min and the signal was detected at 245 nm. The Av standards were used for calibration curve, which were created by diluting a stock solution of 500 μg/mL to 200, 120, 80, 40, 20, 15, 10, 5, 2 μg/mL, respectively. The loading content and entrapment efficiency were calculated using the following equations:

(4) $$LC\left(wt\%\right)=\frac{weight\;of\;pestiside\;in\;microspheres}{weight\;of\;microspheres}\times 100\%$$

(5) $$EE\left(wt\%\right)=\frac{weight\;of\;pestiside\;in\;microspheres}{initial\;weight\;of\;pesticide\;fed}\times 100\%$$

### 2.7. Release Kinetics of Av

To evaluate the release of the entrapped Av from the microspheres, a 4mL of certain amount Av suspension in a methanol/water (40/60, *v*/*v*) mixture was loaded into the dialysis membrane. Then the membrane bag was put into a wild-mouth flask containing 96 mL release media consisting of 40 v% methanol and 60 v% water. The flask was then transferred to the incubator shaker with a speed of 120 rpm at 30 °C. At regular time intervals, 4mL solution was collected and 4 mL fresh solvent was added into the flask back. The concentration of Av released was also determined by HPLC with the same condition above.

## 3. Results and Discussion

### 3.1. Synthesis and Characterization of P(BS-BMS) Copolymers

**Scheme 1:**
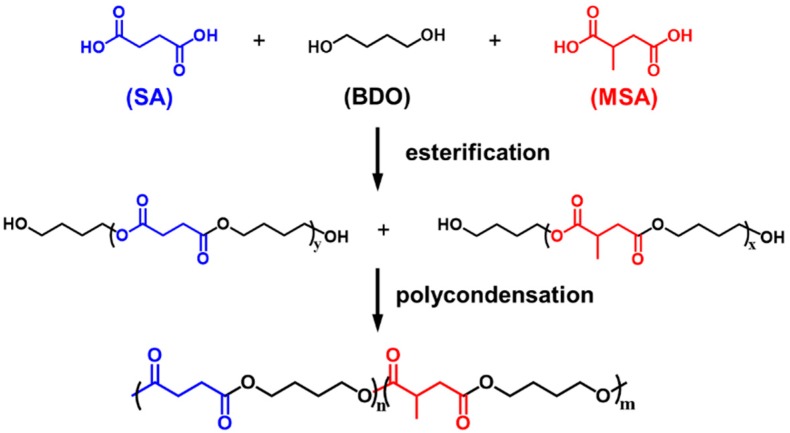
Synthesis route of poly(butylene succinate-co-butylene 2-methylsuccinate) (P(BS-BMS)) copolymers. SA: succinic acid; BDO: 1,4-butanediol; MSA: 2-methylsuccinic acid.

**Figure 1:**
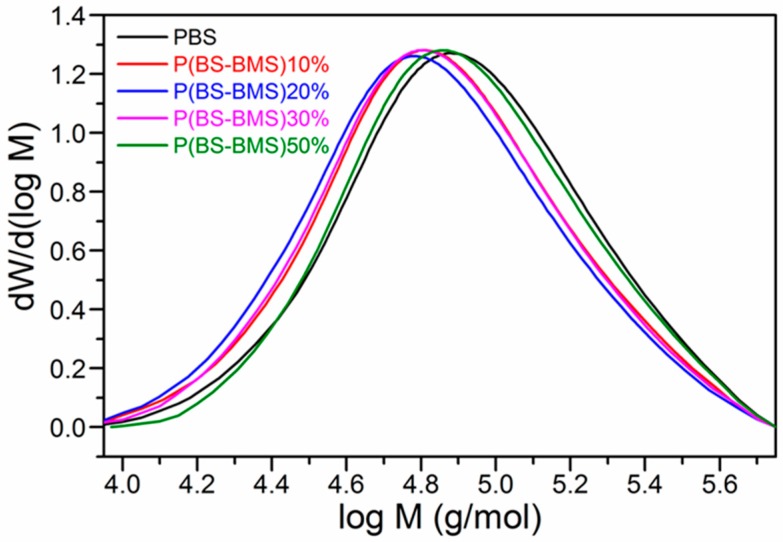
Gel permeation chromatography (GPC) curves of P(BS-BMS) copolymers and pure poly(butylene succinate) (PBS).

MSA was introduced in order to adjust the chain structure of copolymer by a classical two stage procedure as shown in Scheme 1. At first direct esterification stage, BDO could react with both SA and MSA to form ester bonds as the temperature rises. Then the P(BS-BMS) copolymers were produced via further melt polycondensation. The molecular weight and dispersity (Đ) of P(BS-BMS) copolymers are listed in Table 1 and the GPC curves are shown in Figure 1. The P(BS-BMS) copolymers which owned similar *M*_n_ (5.1 × 10^4^–6.4 × 10^4^ g/mol) and Đ (1.64–1.74) were obtained by same synthesis procedure above.

**Figure 2:**
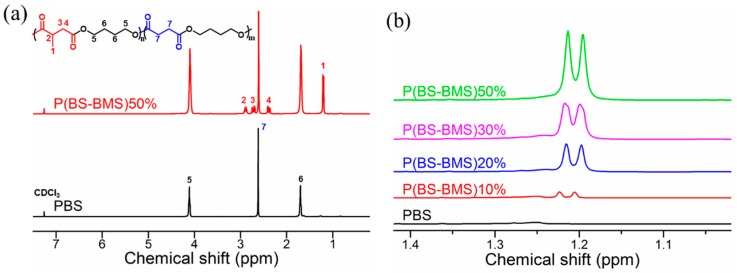
(**a**) ^1^H NMR spectra of pure PBS and P(BS-BMS)50%; (**b**) ^1^H NMR spectra of pure PBS and P(BS-BMS) copolymers at 1.20 ppm.

The ^1^H NMR spectra of P(BS-BMS)50% and pure PBS are shown in Figure 2a. For pure PBS, the peak at 4.10 and 1.69 ppm are attributed to the protons in BDO (δH^5^ and δH^6^) segments, while the chemical shift of CH_2_ in SA (δH^7^) appears at 2.61 ppm. As the proportion of MSA increases, characteristic peaks belonging to MSA (δH^2^, δH^3^, δH^4^, and δH^1^) are detected around 2.88, 2.73, 2.40, and 1.20 ppm, and the increase of δH^1^ intensity is shown in Figure 2b. The actual compositions of P(BS-BMS) copolymers were calculated from the integrated area of peaks at 1.20 ppm (δH^1^, MSA) and 2.61 ppm (δH^7^, SA) according to the following equations:

(6) $$f_{PBS}=\frac{A_{2.61}/4}{A_{1.20}/3+A_{2.61}/4}$$

(7) $$f_{PBMS}=\frac{A_{1.20}/3}{A_{1.20}/3+A_{2.61}/4}$$

where A represents the corresponding peak intensity, and the calculated results are listed in Table 1, as well as the feeding proportion and theoretical content of PBMS. It can be seen that the actual composition is not much different from the feed ratio, indicating that the content of PBMS in copolymers can be accurately controlled by adjusting the feed ratio.

**Figure 3:**
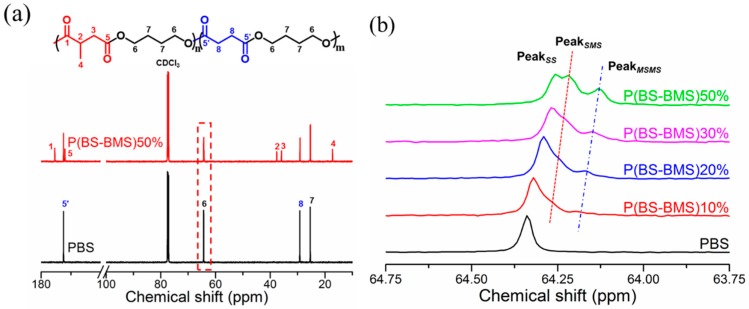
^13^C NMR spectra of (**a**) pure PBS and P(BS-BMS)50%; (**b**) pure PBS and P(BS-BMS) copolymers around 64.3–64.2 ppm attributed to δC^6^. The data were measured in CDCl_3_ in 25 °C.

Generally, the sequence distribution of copolymers has a large influence on the physical and chemical properties. ^1^H NMR and ^13^C NMR spectroscopies can provide detail and quantitative information about nature of linkages and sequence distribution to analyze and verify the relationship between performance and structure of copolymers [60,61,62,63]. When the connection at either sides of target unit changes, the environment of protons and carbon inside the unit are affected and the chemical shift changes to some extent, while the characteristic peak are split into multiple peaks in copolymers. The greater difference in linkage units, the more pronounced the split. In P(BS-BMS) copolymer structures, there are four possible environments for ethylene units in BDO: the homo unit, SS and MSMS, and the hetero unit, SMS and MSS, where S and MS represented the SA and MSA, respectively. Unfortunately, in the ^1^H NMR spectra, the peak split at 4.10 ppm and 1.69 ppm belonging to BDO are not very obvious, owing to the similarity of SA and MSA which makes the hydrogen environment change weakly. ^13^C NMR spectra of P(BS-BMS)50% and pure PBS obtained from the quantify model are shown in Figure 3a, the signals at 172.4 ppm (δC^5^^´^) and 29.1 ppm (δC^8^) are attributed to the carbonyl and methylene in SA unit. As the MSA monomer increased, the new peaks belonging to MSA (δC^1^, δC^2^, δC^3^, and δC^4^) are detected around 175.4, 37.6, 35.9, and 17.2 ppm, respectively. There is also a peak (around 171.9 ppm) next to the chemical shift of the carbonyl peak in the SA unit, which is assigned to the carbonyl carbon away from the methyl group in MSA. In addition, the two peaks appear at 63.3 ppm and 25.4 ppm, which are attributed to the methylene in BDO (δC^6^ and δC^7^). The characteristic peak changes of δC^6^ in P(BS-BMS) copolymers are shown in Figure 3b, the carbon peak split into triple peak around 64.3–64.2 ppm clearly and assigned to SS, SMS(MSS), MSMS, respectively. By the ^1^H NMR spectra analysis and carbon peak split of BDO, it is confirmed that P(BS-BMS) copolymers are synthesized successfully. These integrated resonance intensities (*f*_SS_, *f*_SMS_, *f*_MSMS_) are employed to calculate the sequence length of PBS and PBMS segments and stereoregularity of P(BS-BMS) copolymers as the following equations:

(8) $$L_{n,\mathrm{PBS}}=1+2f_{\mathrm{SS}}/f_{\mathrm{SMS}}$$

(9) $$L_{n,\mathrm{PBMS}}=1+2f_{\mathrm{MSMS}}/f_{\mathrm{SMS}}$$

(10) $$R=1/L_{n,\mathrm{PBS}}+1/L_{n,\mathrm{PBMS}}$$

where *L* represents the sequence length of PBS and PBMS, and *R* represents the degree of randomness, respectively. The calculated results are listed in Table 2, and the *R* values of P(BS-BMS) copolymers are close to 1, which indicates the P(BS-BMS) copolymers with random structure were obtained.

### 3.2. Thermal Properties and Crystal Structures of P(BS-BMS) Copolymers

**Figure 4:**
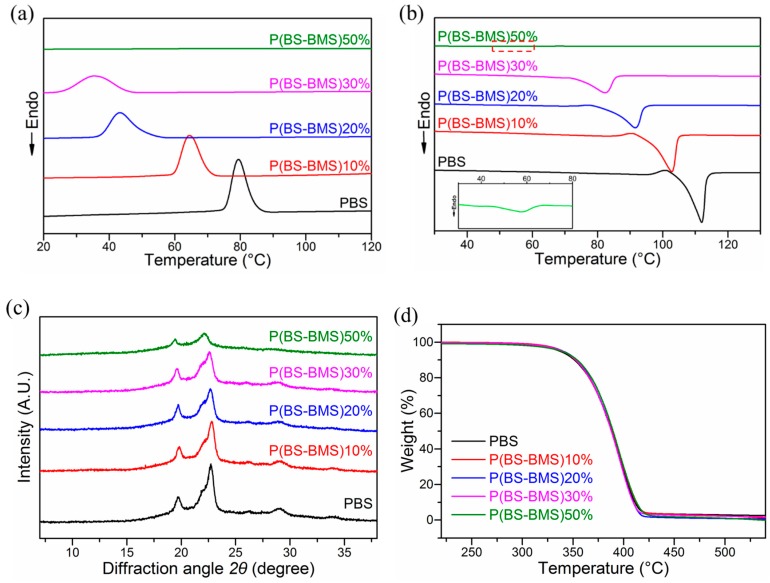
DSC curves of pure PBS and P(BS-BMS) copolymers during (**a**) first cooling from melt at a cooling rate of 10 °C/min and (**b**) second heating process at a heating rate of 10 °C/min (inner figure is magnified second heating curve of P(BS-BMS)50%); (**c**) WAXD patterns of pure PBS and P(BS-BMS) copolymers; (**d**) TGA curves of pure PBS and P(BS-BMS) copolymers at a heating rate of 10 °C/min under N_2_.

The melting and crystallization behavior of copolymer samples were recorded by DSC. The cooling and second heating curves are shown in Figure 4a,b, and the corresponding thermal parameters including melting temperature (*T*_m_), melting enthalpy (*ΔH*_m_), crystallization temperature (*T*_c_) and crystallinity degree (*X*_c_) are listed in Table 3. It can be found that the crystallization behavior of P(BS-BMS) is closely related to the molecular composition and sequence distribution. 

During the cooling procedure, samples with PBMS molar ratio ranging from 0 to 30% are able to crystallize, while P(BS-BMS)50% doesn’t show an exothermic peak which means it maintains an amorphous state at that procedure. On the other hand, former four samples only show a single crystallization peak, which is mainly attributed to crystallization of PBS segment. This crystallinity result is also confirmed by the WAXD test. As shown in Figure 4c, the pure PBS shows three main diffraction peaks at 19.6°, 22.0°, and 22.8°, corresponding to (020), (021), and (110) reflection planes of α-form crystal, respectively. The ratio of PBMS increase to 50%, the samples exhibit three diffraction peaks at the same positions compared to the pure PBS. It means that only PBS segment in the copolymer can crystallize and also MSA does not influence the PBS crystal structure. In addition, pure PBS owns the highest crystallization temperature at 79.4 °C, which gradually decreases in P(BS-BMS) copolymers to 64.6 °C, 43.4 °C, 38.2 °C in P(BS-BMS)10%, P(BS-BMS)20%, and P(BS-BMS)30%, respectively. It indicates that introduction of MSA monomer will weaken the crystallization ability of copolymers because there is a chiral carbon atom attached to the side methyl group in MSA unit.

During the second heating scan, all crystalline samples own single melting point, which shifts from 112.0 °C for pure PBS to 82.4 °C for P(BS-BMS)30% copolymer. As shown in inner curve of Figure 4b, it shows an extremely weak melting behavior at 58.3 °C for P(BS-BMS)50%. These decrease in *T*_m_ and crystallinity are due to the formation of imperfect crystals ascribed to the shortened sequence length of PBS from random copolymerization which indicates the introduction of MSA monomer can result in a significant impact on the melting point and crystallinity.

### 3.3. Thermal Stability and Biodegradation of P(BS-BMS) Copolymers

The thermal stability of P(BS-BMS) copolymers was measured by TGA in nitrogen atmosphere, which is a crucial property for the applicability of aliphatic polyesters. The recorded thermogravimetric curves of pure PBS and P(BS-BMS) copolymers are presented in Figure 4d. And both the decomposition temperature at 5% weight loss (*T*_d,5_) and that at maximum rate (*T*_d,max_) are listed in Table 3. The *T*_d,5_ and *T*_d,max_ of pure PBS and P(BS-BMS) copolymers are ranging from 337.1 to 340.0 °C and 397.6 to 399.4 °C, respectively. It is observed that the introducing of MSA instead of SA hardly affects the thermal stability of P(BS-BMS) copolymers, which means that these copolymers have nearly composition independent *T*_d,5_ and *T*_d,max_. It is mainly because there is no change in the thermal decomposition mechanism while introducing the MSA monomer, which has little influence on the stability of ester bonds and polymer molecular chains.

**Figure 5:**
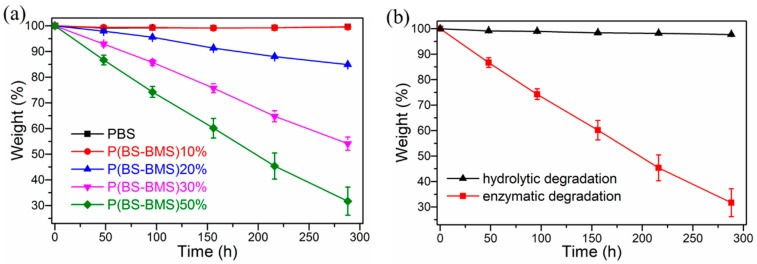
Weight losses of (**a**) pure PBS and P(BS-BMS) copolymer films in enzymatic degradation environment and (**b**) P(BS-BMS)20% films in enzymatic degradation and hydrolytic degradation environment, respectively.

Biodegradability has profound and significant influences for the application of biodegradable materials. In order to investigate the biodegradability of P(BS-BMS) copolymers, the enzymatic degradation experiments were executed with film samples. As shown in Figure 5a, the weight loss ratio of P(BS-BMS) copolymers and pure PBS which were immersed in lipase medium for different time intervals is recorded. It can be observed that the MSA proportion of copolymerization greatly affects the rate of weight loss. As the MSA content increases, the weight loss increases obviously. In the pure PBS and P(BS-BMS)10% samples, there was no significant weight loss after 12 days, as a comparison, when the PBMS content is increasing to 50%, the weight of the sample remained only 31.7% of the original after 12 days. The specific weight loss data of each sample is listed in Table S1. After 12 days, the P(BS-BMS)50% films have been partially broken into pieces, which led that it was hard to be weighed anymore. It is well known that the high amorphous content can promote the degradation rate of polyesters and the initial degradation usually begin in the amorphous phase. It is mainly due to the different ability of the enzyme to bind the active site of amorphous region and crystalline region. The crystalline regions are closed-packed while the amorphous regions are relatively loose. As a result, the amorphous regions are easier for small molecules like water to penetrate and enzyme to attach, which leads to the amorphous regions being more prone to degradation. As a control, the weight loss of the P(BS-BMS)50% sample film in the pure phosphate buffer solution (without lipase) was also investigated. As shown in Figure 5b, unlike the severe degradation of the film in the enzyme phosphate buffer, the weight of the P(BS-BMS)50% sample film doesn’t decrease substantially in the pure phosphate buffer. This result indicates that the P(BS-BMS) copolymer doesn’t degrade rapidly in phosphate buffer without enzyme promotion.

The molecular weight test of P(BS-BMS) copolymers in enzyme degradation solution was also carried out with different time intervals. The detailed data were listed in Table S2. As shown in Figures S1 and S2, the molecular weight of the residual copolymer film shows a decrease trend with time, and the decreasing rate is accelerated as the MSA ratio increases. It can be seen that although the weight of residual PBS film was not obviously reduced in the degradation test, its molecular weight has decreased to the original 92.9% after 12 days.

**Figure 6:**
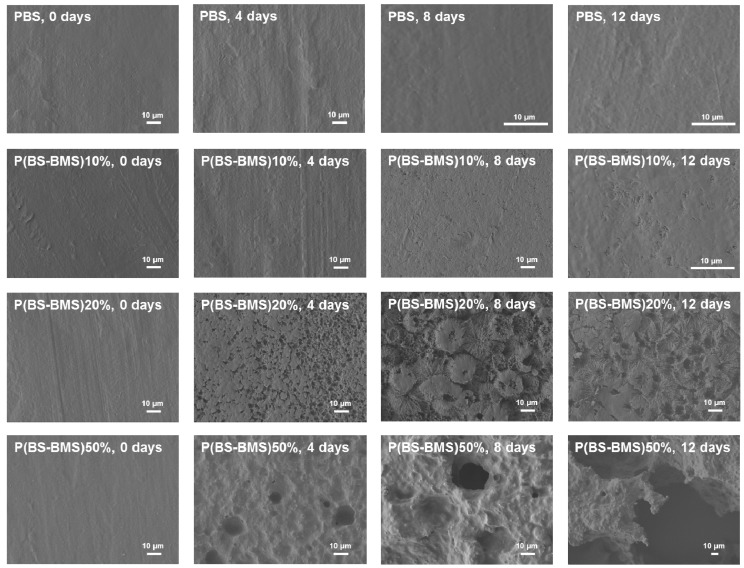
The surface changes of PBS, P(BS-BMS)10%, P(BS-BMS)20%, P(BS-BMS)50% during enzymatic degradation test at different time intervals by SEM.

To gain more insight into the degradation behavior of P(BS-BMS) copolymers, the morphological observation is helpful to understand how the copolymer degrades during the test. Figure 6 presents the surface morphology evolution of the PBS, P(BS-BMS)10%, 20%, 50% film samples during enzymatic degradation process. It shows that the surfaces of all the sample films appear smooth in the beginning. As we can see, during the 12 days of degradation process, there was no significant change in the surface of the PBS film, while for P(BS-BMS)10% film, the surface was slightly roughed after immersed in the enzyme buffer solution, which indicates that the P(BS-BMS)10% sample had slight degradation with the unobvious weight loss. Conversely, for P(BS-BMS)50% film sample, the surface of sample film became rough quickly and many small holes ranging from 1 to 10 μm appeared. Further, the pores were enlarged to form small fragments which contained both amorphous and crystalline regions removing from the film in the degradation of the enzyme buffer solution, and finally, the sample was no longer a whole and became into pieces. For P(BS-BMS)20%, we could observe more details of the degradation process. After the corrosion of the enzymatic degradation solution, the clear spherulites presented. It proves that the degradation rate of the amorphous regions is much faster than that of the crystallization regions, which ultimately leads to the retention of a relatively complete cluster-like morphology of crystallization regions. From the above, the SEM observations are consistent with the weight loss results, indicating that the increase of PBMS content can accelerate the rate of enzyme degradation. From the results of the above enzymatic degradation test showed that for P(BS-BMS), a fully biodegradable material, we could control the degradation rate of P(BS-BMS) copolymers by changing the content of the MSA.

### 3.4. Preparation of the Av-Loaded Delivery Systems Based on P(BS-BMS) Copolymers

**Scheme 2:**
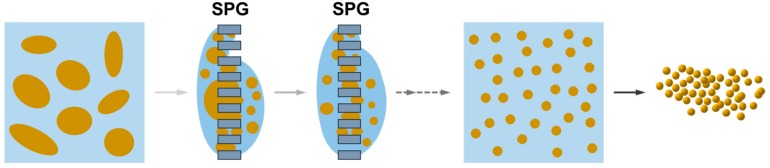
Preparation of pesticide-loaded microparticles process by premix membrane emulsification (PME) method. SPG: Shirasu Porous Glass membrane.

Carrier particle size has a great influence on drug release rate. Many studies have shown that as the particle size of drug-carrying microspheres decreases, the surface area increases, and small molecules are easy to pass through the carrier, leading to the increase of drug release rate. Besides, excellent particle size uniformity and dispersion can achieve efficient drug utilization and controlled release. As illustrated in Scheme 2, the PME method was used to prepare Av-loaded microspheres based on P(BS-BMS) copolymers by using SPG membrane with pore size of 5.4 μm. Compared with mechanical stirring, this method can efficiently obtain products with uniform sizes. As listed in Table 4, the Av-loaded microparticles based on different copolymers were obtained with actual size ranging from 1.31 μm to 1.84 μm with narrow size distribution (*PDI* < 1.3). No heating in the entire preparation process is another advantage of PME method, which avoids degradation of pesticides under high temperature conditions. Submicron-level pesticide formulations can improve the dispersibility of poorly soluble pesticide molecules and enhance the effective utilization of the pesticide.

**Figure 7:**
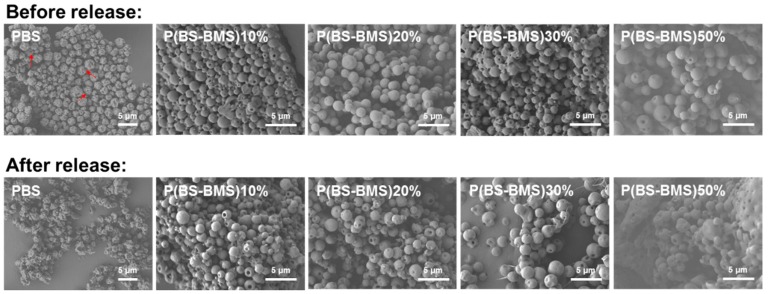
Morphology of different Av-loaded microparticles before and after release in 240 h.

The morphology of the microparticles is another important factor affecting the drug release rate. The morphology of Av-loaded microparticles based on P(BS-BMS) copolymer and pure PBS was observed by SEM as shown in Figure 7. As we can see in Figure 7 (the upper row), all the Av-loaded microparticles based on P(BS-BMS)10%–50% exhibit an approximately spherical shape with some small holes on the surface. These defects on the surface mainly attribute to the phase separation during the solvent evaporation process. Unlike the above samples, the morphology of Av-loaded microparticles based on pure PBS exhibits an irregular shape with multiple folds on the surface structure instead of smooth spherical microparticles (as indicated by the red arrows). In the solvent evaporation and phase separation process, since the solubility of the pure PBS in chloroform is much lower than that of the P(BS-BMS) copolymer, the PBS system separates out from the organic phase earlier. The initial formed PBS shell is too thin to support the formation of microparticles, and then collapses to form irregular microparticles with large wrinkles. In addition, the irregular shape with rough surface is another advantageous for the retention of the Av-loaded system on the leaves compared to other systems with the intact spherical shape. The depression on the surface of microparticle also helps the Av-loaded microparticles to hang on the fluff on the surface of the leaves. 

### 3.5. Av entrapment Efficiency and Encapsulation State

Loading content and entrapment efficiency are two important indicators for evaluating overall performance pesticide release systems. The high drug loading and entrapment efficiency not only reduces the waste of active ingredients during the preparation process but also reduces environmental pollution and personal injury caused by excessive use during actual application. The loading content and entrapment efficiency of all formulations are listed in Table 4, which indicates that the entrapment efficiency of each form is higher than 67%. As the crystallization ability of the P(BS-BMS) copolymers decreases, the entrapment efficiency of the Av ingredient approximately tends to decrease. It mainly because the crystallization regions can limit the diffusion of the Av molecules resulting in being trapped in microparticles during the solvent evaporation process.

**Figure 8:**
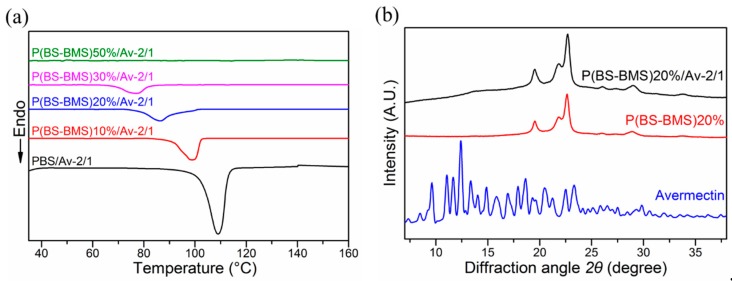
(**a**) DSC curves of Av-loaded microparticles based on pure PBS and P(BS-BMS) copolymers during first heating process at a rate of 10 °C/min; (**b**) WAXD patterns of Avermectin, P(BS-BMS)20% and P(BS-BMS)20%/Av-2/1, respectively.

The DSC and wide angle X-ray diffraction test were used to detect the physical state of the Av-loaded systems including carrier polymers and active ingredients. As shown in Figure 8a, the DSC results shows only the PBS segment melting peak appears in the first heating curve of the Av-loaded microparticles, which means that the pesticide molecules were dispersed in the polymer in an amorphous state. Furthermore, the melting peak of the PBS crystal moves to the low temperature region and the melting range becomes wider, which indicates that the introduction of the pesticide molecules affects the PBS segments into the lattice. As shown in Figure 8b, a large number of sharp diffraction peaks are detected with crystalline Av molecules. As a comparison, these characteristic peaks attributed to Av were not detected in P(BS-BMS)20%/Av-2/1 system, whereas only the characteristic diffraction peaks belonging to PBS segment were revealed. This is consistent with the above DSC results, the introduction of the Av molecules does not change the crystalline form of the P(BS-BMS) matrix.

### 3.6. Control Release of Av

**Figure 9:**
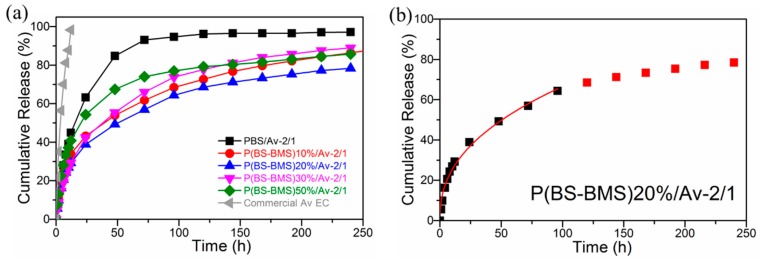
(**a**) the release behavior of commercial Av form and Av-loaded microparticles based on different P(BS-BMS) copolymers; (**b**) fitting curve of P(BS-BMS)20%/Av-2/1 release profile according to Korsmeyer–Peppas equation.

In order to study the pesticide release mechanism, the release behavior of Av-loaded microparticles based on different P(BS-BMS) copolymers and pure PBS was investigated. As shown in Figure 9a, compared to Av commercial emulsion formulations, all microparticle forms exhibit a relatively slow release rate, which means that in the actual application process, it can achieve long-term stable release and realize the purpose of reducing the pesticides usage amount and microplastic pollution. Additionally, all the drug release process presents an initial burst release at first followed by a sustained release for longer period. During the initial release process, the Av active ingredients which are adsorbed in the surface layer of the microparticles can be dissolved in the release environment quickly by directly contacting the solvent, resulting in the rapid release process. After that, the Av molecules inside the microparticles slowly diffuses into the medium over time. The Korsmeyer-Peppas equation is considered to fit the Av-release data in order to analyze changes in the drug release process system:

(11) $$\frac{Q_{t}}{Q_{\infty }}=kt^{n}$$

where the QQ∞ is the Av release ratio, *k* is a kinetic constant, *t* is the release time and the n is diffusion exponent which indicates the drug release mechanism. For sphere-form system, *n* = 0.43 means Fick diffusion and higher values of n, between 0.43 and 0.85 or *n* = o.85 mean mass transfer like dynamic swelling or degradation of matrix following a non-Fickian model. It must note that the part with the QtQ∞<0.6 of the release curve should only be used for determination of the exponent *n*. The fitting curve of P(BS-BMS)20%/Av-2/1 microparticles release process according to *Korsmeyer–Peppas* equation is shown in Figure 9b (other fitting curves based on P(BS-BMS) copolymers and pure PBS are shown in Figures S3–S6, respectively), and more detailed parameters are listed in Table 4. The correlation coefficient (*r*^2^) of all samples is higher than 0.97 which means the release curve matched well with the Korsmeyer-Peppas equation. For Av-loaded microparticles based on P(BS-BMS)10%, P(BS-BMS)20% and P(BS-BMS)30%, *n* is nearly 0.43, which indicates that the Av release basically follows Fick diffusion in these systems. In other two systems, *n* slightly larger than 0.43 illustrates some changes of polymer matrix accelerate the diffusion rate of Av molecules except following the Fick diffusion. In order to better explain the changes in the matrix, the morphology of the Av-loaded microparticles after drug release was also observed by SEM. As shown in Figure 7 (the lower row), for the Av-loaded system based on pure PBS matrix, further structural collapse and damage of the microparticles can be clear observed, which can promote the penetration of the release solution and the rate of Av release. As for P(BS-BMS)50%, the change of morphology is different from the PBS/Av system. In this systems, the microparticles adhered to each other during release process. This indicates that due to the increasing proportion of amorphous regions in the polymer matrix, a portion of the matrix can swell or deform in the release medium to accelerate the Av-release rate during the release of Av.

Therefore, the crystallinity of the matrix is a key factor affecting the rate of drug release. For high crystallinity polymers like pure PBS, folded-surface morphology can be formed during the preparation process. This morphology increases the surface area met with the release medium, besides during the release process, the loose particulate structure tends to collapse even damage and thereby promote drug release. As for high amorphousness polymers, diffusion of the active ingredient is much easier in the amorphous region owing to higher mobility of molecular chains in amorphous state than crystal state in the release medium. Additionally, much easier penetration of release medium through the amorphous regions also accelerates the drug release rate. A relatively slow release drug system can be obtained based on a polymer matrix with proper crystallinity. The lamellae structure may limit the diffusion of small molecules and chain movement attributed to its poor motion ability which leads to prolonged release pathway of Av molecules.

## 4. Conclusions

A series of P(BS-BMS) copolymers were successfully prepared via direct two-step esterification and polycondensation method. The P(BS-BMS) copolyesters presented widely tunable properties including melting temperature and degree of crystallinity, ranging from high crystallinity plastics to flexible amorphous polymers. As the MSA content increased, the proportion of the amorphous region of the copolymer increased gradually, which made the copolymer easier for microorganisms and degrading enzymes to adhere to the reaction sites, resulting in a significant acceleration in biodegradability. This was mainly because the side methyl group in the MSA unit destroyed the regularity of the segment and prevented the segment from being discharged into the crystal lattice. At the same time, the thermal stability of P(BS-BMS) copolymers showed no obvious change with the increasing of MSA content. Then we successfully prepared Av-loaded microparticles based on P(BS-BMS) copolymers with uniform size by PME method and these Av-loaded systems showed an initial burst release followed by a significantly sustained release over 10 days in a methanol/water (40/60, *v*/*v*) mixture. The effects of crystallinity on the morphology of microparticles and drug release profile were also discussed, which showed the proper crystallinity could be used to control release kinetics to meet practical requirements of drug release. It was worth mentioning that the P(BS-BMS) copolymers could be completely biodegraded so as not to bring additional micro-plastic pollution pressure to the water and soil environment when they were applied to agriculture field.

## Figures and Tables

**Table 1:** Composition and molecular weight of P(BS-BMS) copolymers and pure PBS.

Polymer	MSA (mol)	SA (mol)	BDO (mol)	Theoretical Content of PBMS (%)	Experimental Content of PBMS (%)	*M*_n_ (10^−4^ g/mol)	*M*_w_ (10^−4^ g/mol)	*Đ*
**PBS**	-	0.2	0.24	0	0	6.3	10.7	1.70
**P(BS-BMS)10%**	0.02	0.18	0.24	10.0	9.5	5.5	9.5	1.73
**P(BS-BMS)20%**	0.04	0.16	0.24	20.0	20.3	5.1	9.0	1.76
**P(BS-BMS)30%**	0.06	0.14	0.24	30.0	29.1	5.5	9.4	1.71
**P(BS-BMS)50%**	0.1	0.1	0.24	50.0	48.5	6.4	10.5	1.64

**Table 2:** Sequence distribution analysis of P(BS-BMS) copolymers.

Copolymer	*f*_SS_ (%)	*f*_SMS_ (%)	*f*_MSMS_ (%)	*L*_n,S-BDO_	*L*_n,MS-BDO_	*R*
**P(BS-BMS)10%**	77.3	19.4	3.3	9.0	1.3	0.88
**P(BS-BMS)20%**	53.7	36.6	9.7	3.9	1.5	0.92
**P(BS-BMS)30%**	43.9	40.3	15.9	3.2	1.8	0.87
**P(BS-BMS)50%**	32.2	47.6	20.2	2.3	1.9	0.96

**Table 3:** Thermal parameters of pure PBS and P(BS-BMS) copolymers.

Sample	*T*_m_ (°C)	*ΔH*_m_ (J/g)	*T*_c_ (°C)	*X*_c_ (%)*^a^*	Thermal Degradation (°C)	
					*T*_d,5_	*T*_d,max_
**PBS**	112.0	64.3	79.4	58.2	337.7	398.3
**P(BS-BMS)10%**	102.8	54.2	64.6	49.1	340.0	397.6
**P(BS-BMS)20%**	91.6	48.0	43.4	43.5	339.9	399.4
**P(BS-BMS)30%**	82.4	41.0	35.4	37.1	339.5	398.1
**P(BS-BMS)50%**	58.3	0.2	-	0.2	337.1	399.1

*^a^* The degree of crystallinity (*X*_c_) was calculated from the DSC results, *ΔH*_0_ (PBS)=110.4 (J/g) [31].

**Table 4:** Parameters of pesticide-loaded microparticle size and release performance.

Sample	Size (μm)	*PDI*	Theoretical Loading Content (%)	Measured Loading Content (%)	Entrapment Efficiency (%)	*n*	*r^2^*
**PBS/Av-2/1**	1.84	1.24	33.3	26.6	79.9	0.62	0.99
**P(BS-BMS)10%/Av-2/1**	1.32	1.27	33.3	28.2	84.7	0.40	0.98
**P(BS-BMS)20%/Av-2/1**	1.31	1.24	33.3	29.5	88.5	0.42	0.99
**P(BS-BMS)30%/Av-2/1**	1.54	1.12	33.3	26.2	78.7	0.50	0.99
**P(BS-BMS)50%/Av-2/1**	1.61	1.21	33.3	22.5	67.6	0.45	0.97

## Supplementary Materials

The following are available online at https://www.mdpi.com/1996-1944/12/9/1507/s1, Figure S1: *M*_n_ losses of pure PBS and P(BS-BMS) copolymer residual films in enzymatic degradation environment in different time intervals. Figure S2: GPC curves of P(BS-BMS) copolymers and pure PBS after 12 days degradation process. Figure S3: Fitting curve of P(BS-BMS)10%/Av-2/1 release profile according to Korsmeyer-Peppas equation. Figure S4: Fitting curve of P(BS-BMS)30%/Av-2/1 release profile according to Korsmeyer-Peppas equation. Figure S5: Fitting curve of P(BS-BMS)50%/Av-2/1 release profile according to Korsmeyer-Peppas equation. Figure S6: Fitting curve of PBS/Av-2/1 release profile according to Korsmeyer-Peppas equation. Table S1: Weight loss data of pure PBS and P(BS-BMS) copolymers during enzyme degradation test. Table S2: Molecular weight data of pure PBS and P(BS-BMS) copolymers by GPC test.
