# Supplementary material for: Synthesis, Properties of Biodegradable Poly(Butylene Succinate-co-Butylene 2-Methylsuccinate) and Application for Sustainable Release
Source: Materials (Basel). 2019 May 9;12(9):1507. doi: 10.3390/ma12091507 (PMC6539853; doi:10.3390/ma12091507)
Supplement: Supplementary file 1 [file materials-12-01507-s001.pdf]

materials

Supplementary Materials

Synthesis, Properties of Biodegradable Poly(Butylene Succinate-*co*-Butylene 2-Methylsuccinate) and Application for Sustainable Release

Jiarui Han, Jiaxin Shi, Zhining Xie, Jun Xu * and Baohua Guo *

Key Laboratory of Advanced Materials of Ministry of Education of China, Department of Chemical Engineering, Tsinghua University, Beijing 100084, China; chaxiangmeimei@163.com (J.H.); shijx13@tsinghua.org.cn (J.S.); xiezhining@126.com (Z.X.)

* Correspondence: jun-xu@mail.tsinghua.edu.cn (J.X.); bhguo@mail.tsinghua.edu.cn (B.G.)

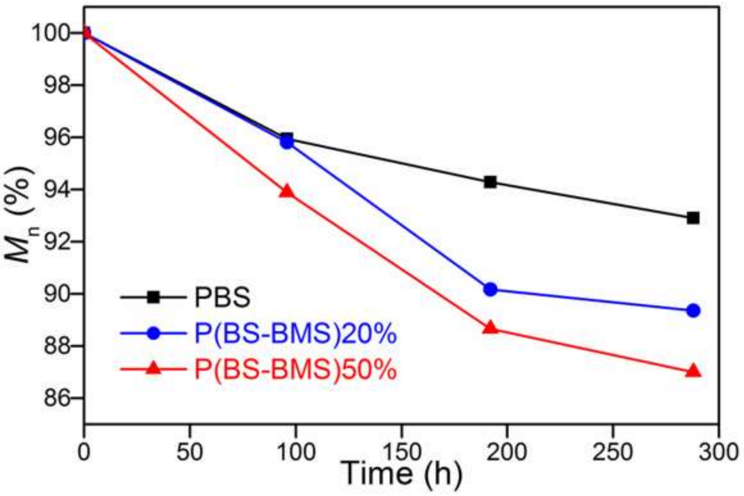

Figure S1. M_n losses of pure PBS and P(BS-BMS) copolymer residual films in enzymatic degradation environment in different time intervals.

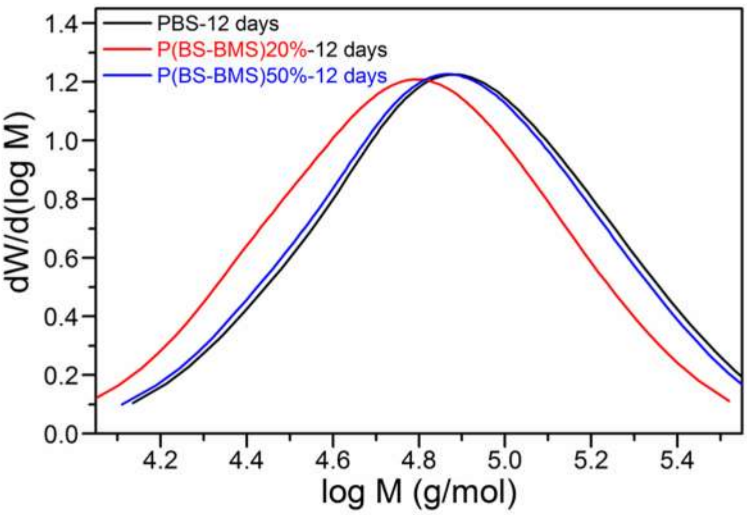

Figure S2. GPC curves of P(BS-BMS) copolymers and pure PBS after 12 days degradation process.

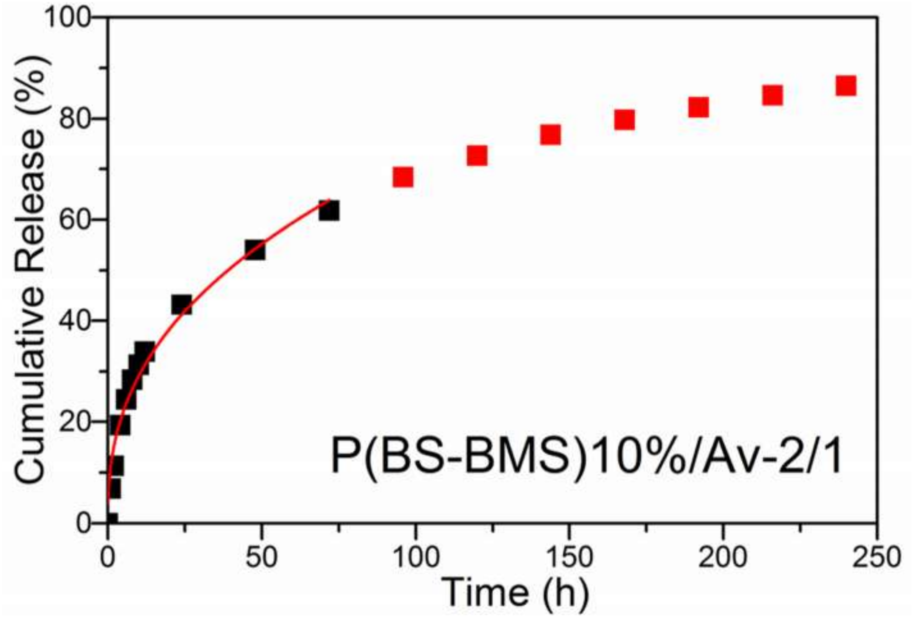

Figure S3. Fitting curve of P(BS-BMS)10%/Av-2/1 release profile according to *Korsmeyer-Peppas* equation.

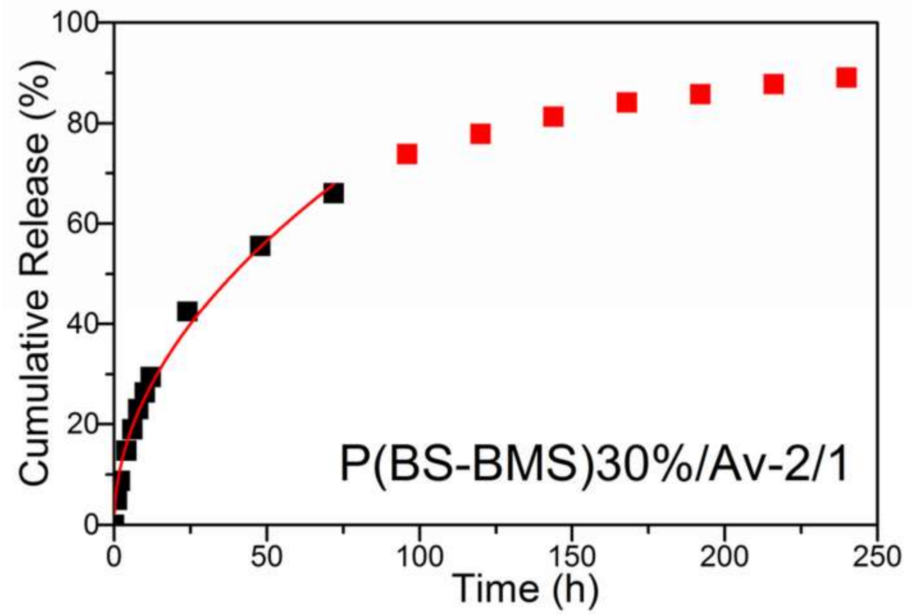

Figure S4. Fitting curve of P(BS-BMS)30%/Av-2/1 release profile according to *Korsmeyer-Peppas* equation.

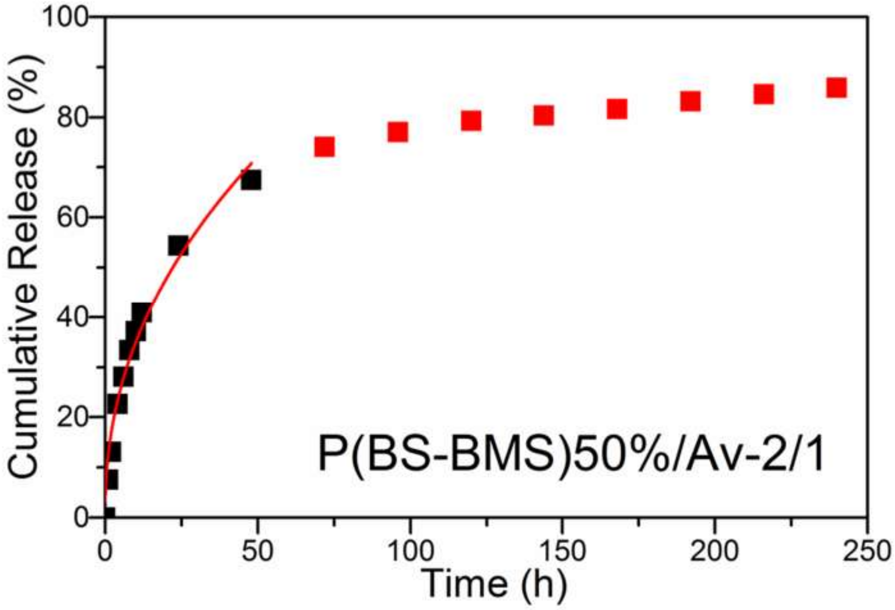

Figure S5. Fitting curve of P(BS-BMS)50%/Av-2/1 release profile according to *Korsmeyer-Peppas* equation.

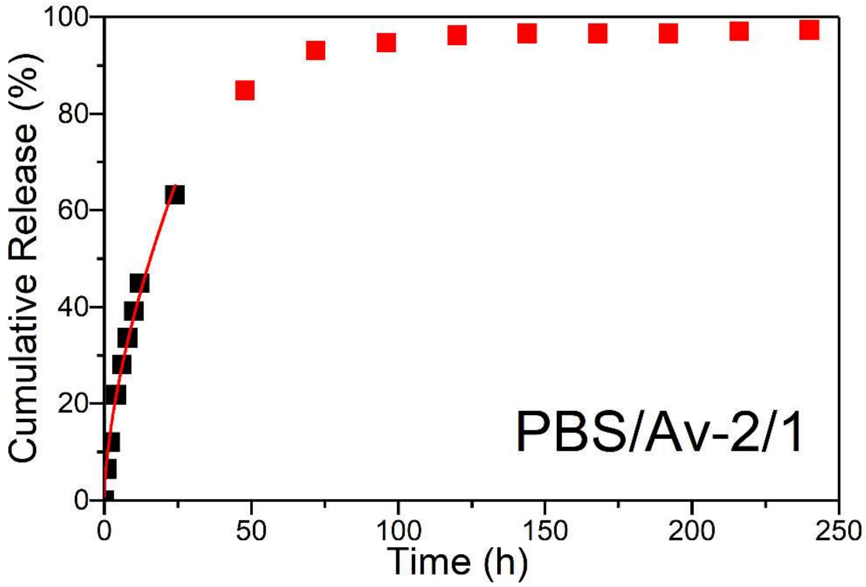

Figure S6. Fitting curve of PBS/Av-2/1 release profile according to *Korsmeyer-Peppas* equation.

Table S1. Weight loss data of pure PBS and P(BS-BMS) copolymers during enzyme degradation test.

Copolymer	Initial (%)	48h (%)	96h (%)	156h (%)	216h (%)	288h (%)
**PBS**	100	99.1	99.2	99.1	99.2	99.5
**P(BS-BMS)10%**	100	99.3	99.2	99.1	99.2	99.5
**P(BS-BMS)20%**	100	97.8	95.5	91.2	88.0	84.8
**P(BS-BMS)30%**	100	92.8	85.7	75.7	64.7	54.1
**P(BS-BMS)50%**	100	86.7	74.2	60.1	45.3	31.6

Table S2. Molecular weight data of pure PBS and P(BS-BMS) copolymers by GPC test.

**Copolymer**	**4 days**		**8 days**		**12 days**	
	**M_n×10^{-4} (g/mol)**	**PDI**	**M_n×10^{-4} (g/mol)**	**PDI**	**M_n×10^{-4} (g/mol)**	**PDI**
**PBS**	6.04	1.71	5.94	1.72	5.85	1.74
**P(BS-BMS)20%**	4.92	1.69	4.63	1.76	4.59	1.76
**P(BS-BMS)50%**	6.04	1.71	5.70	1.81	5.60	1.75

© 2019 by the authors. Licensee MDPI, Basel, Switzerland. This article is an open access article distributed under the terms and conditions of the Creative Commons Attribution (CC BY) license (http://creativecommons.org/licenses/by/4.0/).
